# Concentrations versus amounts of biomarkers in urine: a comparison of approaches to assess pyrethroid exposure

**DOI:** 10.1186/1476-069X-7-55

**Published:** 2008-11-04

**Authors:** Marie-Chantale Fortin, Gaétan Carrier, Michèle Bouchard

**Affiliations:** 1 Department of Environmental and Occupational Health, Université de Montréal, P.O. Box 6128, Main Station, Montreal, Quebec, H3C 3J7, Canada

## Abstract

**Background:**

Assessment of human exposure to non-persistent pesticides such as pyrethroids is often based on urinary biomarker measurements. Urinary metabolite levels of these pesticides are usually reported in volume-weighted concentrations or creatinine-adjusted concentrations measured in spot urine samples. It is known that these units are subject to intra- and inter-individual variations. This research aimed at studying the impact of these variations on the assessment of pyrethroid absorbed doses at individual and population levels.

**Methods:**

Using data obtained from various adult and infantile populations, the intra and inter-individual variability in the urinary flow rate and creatinine excretion rate was first estimated. Individual absorbed doses were then calculated using volume-weighted or creatinine-adjusted concentrations according to published approaches and compared to those estimated from the amounts of biomarkers excreted in 15- or 24-h urine collections, the latter serving as a benchmark unit. The effect of the units of measurements (volume-weighted or creatinine adjusted concentrations or 24-h amounts) on results of the comparison of pyrethroid biomarker levels between two populations was also evaluated.

**Results:**

Estimation of daily absorbed doses of permethrin from volume-weighted or creatinine-adjusted concentrations of biomarkers was found to potentially lead to substantial under or overestimation when compared to doses reconstructed directly from amounts excreted in urine during a given period of time (-70 to +573% and -83 to +167%, respectively). It was also shown that the variability in creatinine excretion rate and urinary flow rate may introduce a bias in the case of between population comparisons.

**Conclusion:**

The unit chosen to express biomonitoring data may influence the validity of estimated individual absorbed dose as well as the outcome of between population comparisons.

## Background

The assessment of human exposure to environmental contaminants such as chemical pesticides is often based on the measurement of metabolites excreted in urine. In such studies, participants are asked to provide either a spot sample, multiple spot samples or pooled voids over timed collection periods (ex: 24 h). Biomarker results can therefore be expressed in terms of volume-weighted concentrations (μg/L or nmol/L, for example), creatinine-adjusted concentrations (μg/g of creatinine or μmol/mol of creatinine), cumulative amounts of metabolite excreted over specified periods of time, usually 24 h (μg/day or nmol/day), or hourly excretion rates (ng/h or pmol/h). All those units can be corrected for the body weight of the participants.

In the specific case of non-persistent pesticides, namely pyrethroids and organophosphates (OPs), the determination of amounts of a biomarker in 24-h urine collections is generally considered the "benchmark" unit of expression of biomonitoring results [1-3]. Some authors have successfully measured cumulative daily amounts or hourly excretion rates of pyrethroids (μg or nmol/day or ng or pmol/h) [4-8] and a few others are available in the case of OP pesticides [6,9-11]. However, it is often deemed difficult to achieve in some cases due the risk of incomplete samples, insufficient adherence to the protocol and potential attrition among the participants.

Therefore, urinary metabolite levels of these pesticides are usually reported in volume-weighted concentrations (μg/L or nmol/L; [12-20]) and/or creatinine-adjusted concentrations (μg/g of creatinine; [15,21-24]). Such units are generally used when participants provide one or more spot samples. However, the use of spot urine samples has been questioned in the case of some non-persistent pesticides [25,26].

Volume-weighted concentrations are influenced by urine dilution, which has been documented to be subject to important intra- and inter-individual variations due to the variability in intrinsic and extrinsic factors: liquid intake, perspiration, humidity level, circadian changes, glomerular filtration rate, age, disease (e.g., diabetes, lupus), menstrual cycle, etc. [27-30]. To account for the variations in urine volume, numerous researchers rely on creatinine standardization although creatinine levels have also been reported to be subject to intra- and inter-individual variations linked to: physical activity, circadian changes, age, gender, ethnicity, body size, lean-body mass and protein intake [29,31-36].

Creatinine-adjusted concentrations or volume-weighted concentrations of pesticide metabolites have been used to estimate the absorbed daily dose in individuals [37-40]. Creatinine-adjusted concentrations are thus extrapolated to 24-h excretions using age- and gender-specific default daily creatinine excretion values taken from the biomedical literature or using calculated values which also consider height and weight. As for volume-weighted concentrations, they are converted to 24-h excretions using age- and gender-specific default daily urine volumes.

Pyrethroids are compounds with relatively short half-lives and a large fraction, as much as 78%, of an oral dose in volunteers is excreted as metabolites in urine within 24 hours post-exposure [41]. Therefore, the time of sampling could influence the outcome of the extrapolation to 24 h, as in the case of OP pesticides for which it has been reported that a first morning void should be preferred if only a spot sample is collected [42]. The influence of the toxicokinetics of non-persistent pesticides on the choice of a sampling time frame was more thoroughly discussed in Barr et al. [26]. However, the influence of the variability in creatinine excretion rate and urinary flow rate on estimated absorbed doses reconstructed from creatinine-adjusted concentrations or volume-weighted concentrations has not been systematically assessed in the case of the non-persistent pyrethroid pesticides.

At the population level, the variability in creatinine excretion rate may also influence results of comparisons of creatinine-adjusted concentrations of biomarkers between studies while the variability in the urinary flow rate may affect volume-weighted concentrations. This variability may have an impact on results of comparisons of biomonitoring data between populations, under different units of measurements, but this has yet to be verified.

The purpose of this study was to determine the relative impact of the different units of expression of pyrethroid biomarker data (volume-weighted concentrations, creatinine-adjusted concentrations and amounts of metabolite excreted) i) on estimates of reconstructed absorbed doses and ii) on results of comparisons of biomonitoring data between studies while further documenting intra- and inter-individual variability in creatinine excretion rate and urinary flow rate.

## Methods

### Study groups and sample collection

For the determination of the impact of units of expression of biomarker data on exposure assessment, data from three pesticide biomonitoring studies were used. Only the participants who achieved a full collection during the required period (i.e. did not report urine losses of more than a few drops) were considered for the present analysis. All studies were approved by the Human Research Ethics Committee of the Faculty of Medicine of the Université de Montréal. Adult participants gave their informed consent and children gave their assent in addition to the parents consent.

Study 1 consisted in the biomonitoring of OP exposure in a group of horticultural greenhouse workers with low physical workload (n = 15) [9,43]. The workers collected all their urine during a 24-h period; they provided each void in a separate bottle and recorded the time of each micturition. Characteristics of the participants are shown in Table 1.

**Table 1:** Characteristics of the subjects in study 1

Subject	Gender	Weight (kg)
Subject 1	Male	75
Subject 2	Male	64
Subject 3	Female	57
Subject 4	Female	48
Subject 5	Female	43
Subject 6	Female	55
Subject 7	Female	50
Subject 8	Male	77
Subject 9	Female	64
Subject10	Female	56
Subject 11	Female	50
Subject 12	Female	68
Subject 13	Male	73
Subject 14	Female	55
Subject 15	Female	57

Study 2 was conducted in the adult and infantile general population of a large urban and suburban area, the Montreal Island, Quebec, Canada [4]. Adults (n = 74) collected all their urine during a period of 24 h, in two separate bottles. The first bottle contained the urines voided between 6 pm until the next morning including first urine void on rising. In the second bottle, all the urines voided following the first morning void until 6 pm were cumulated. Children (n = 74) only carried out the overnight collection from 6 pm until the next morning including first morning void (equivalent to the first bottle in adults).

Study 3 was conducted in the adult and infantile general population of the large rural and agricultural area of the Montérégie, Quebec, Canada [5]. Adults (n = 109) and children (n = 42) collected in one bottle all their urine voided overnight from 6 pm until the next morning including first void upon waking. Characteristics of the participants of studies 2 and 3 are depicted in Table 2.

**Table 2:** Characteristics of the subjects in studies 2 and 3

Variable	Study 2 (Urban)		Study 3 (Rural)	
		
	Adults	Children	Adults	Children
**N**	74	74	109	42
**Gender**				
n (%) female	51 (69)	37 (50)	57 (52)	20 (48)
**Age (years)**				
Range	22–63	6–12	18–70	5–12
Median	41	9	48	8
5^th^, 95^th ^percentiles	26–60	7–12	26–64	6–12
**Weight (kg)**				
Range	38–103	18–61	50–127	15–73
Median	66	32	73	27
5^th^, 95^th ^percentiles	47–92	22–54	53–106	20–51
**Height (m)**				
Range	1.50–1.93	1.04–1.73	1.47–1.93	1.00–1.73
Median	1.66	1.40	1.68	1.32
5^th^, 95^th ^percentiles	1.55–1.82	1.15–1.57	1.53–1.84	1.12–1.63

In both studies 2 and 3, subjects were asked to carefully note the exact time of urine collection (first and last urine in each bottle) and the time of the last urination prior to the onset of urine collection. This allows the precise determination of the accumulation time in the bladder and, hence, the determination of the urinary flow rate, creatinine excretion rate and metabolite excretion rates.

### Urine analysis

Samples were analyzed at the *Laboratoire de Toxicologie Humaine *of the *Institut national de santé publique du Québec*. This public laboratory takes part in an international inter-laboratory testing program for quality assurance.

Among the different parameters measured and of interest in the current work, in study 1, it included the measurement of total urine volume and of creatinine contents. In studies 2 and 3, it included total urine volume and creatinine contents as well as the following pyrethroid metabolites: 3-phenoxybenzoic acid (PBA), cis-3-(2,2-dichlorovinyl)-2,2-dimethylcyclopropane carboxylic acid (cDCCA) and trans-3-(2,2-dichlorovinyl)-2,2-dimethylcyclopropane carboxylic acid (tDCCA). The creatinine concentration was assessed with a DRI creatinine kit from Diagnostix Ltd (Mississauga, Canada). The other analytical methods followed are described in details elsewhere [4,5,9,43].

### Data analysis

#### Assessment of the variability in the urinary flow rate and creatinine excretion rate

Metabolite concentrations (μg/L) depend on urine dilution and creatinine-adjusted metabolite concentrations (μg/g of creatinine) depend on creatinine excretion rate. To assess urine dilution, the urinary flow rate, which corresponds to the volume of urine excreted on average per unit of time (mL/h), was calculated. To evaluate creatinine excretion, the average creatinine excretion rate (μg/h) was computed rather than creatinine concentration because the latter is influenced by urine dilution. The urinary flow rate and creatinine excretion rate were determined for each micturition in the case of the participants of study 1 and for each collection period in the case of the participants of studies 2 and 3.

From study 1, the void-to-void intra-individual variability in the urinary flow rate and creatinine excretion rate was assessed over a 24-h period. For each subject, the following descriptive statistics were computed in Microsoft Excel (Office 2000, Microsoft corp.): the mean ($\bar{\text{x}}$), the minimum (MIN) and maximum (MAX) rates, the maximum to minimum rate ratio (MAX/MIN), and the coefficient of variation in rate values (expressed as a percentage (%CV)).

From studies 2 and 3, the inter-individual variability in the hourly urinary flow rate and creatinine excretion rate (established on the basis of nighttime collections) was evaluated within each study and in both studies combined. In all cases, adults and children were analyzed separately. Descriptive statistics were computed in Excel ($\bar{\text{x}}$, MIN, 5^th ^and 95^th ^percentiles, MAX, MAX/MIN and %CV). Distributions of the 12-h volumes and 12-h creatinine excretion were also established for the adult and infantile urban and rural populations.

#### Effect of the variability in the urinary flow rate and creatinine excretion rate on individual exposure assessment

Once the variability in the urinary flow rate and creatinine excretion rate was determined in the studied population samples, its influence on estimates of reconstructed absorbed doses from volume-weighted concentrations or creatinine-adjusted concentrations was assessed. More specifically, estimates of absorbed doses reconstructed from biomarker concentrations were calculated for each individual within each of the studied populations (adult and children separately and studies 2 and 3 also separately). Those concentration-based dose estimates were compared to doses individually reconstructed from cumulative amounts of pyrethroid metabolites in nighttime urine collections, which was considered as the "standard" approach.

##### Estimation of daily absorbed dose from cumulative amounts of metabolite found in nighttime collections

For dose reconstruction, molar amounts of metabolites excreted during the nighttime collection period (more or less 15 h) were extrapolated to 24 hours by calculating the hourly excretion rate (MHER, equation 1) and by multiplying its value by 24 and by the molecular weight of permethrin (MW_p_) ((DDCAp) equation 2). The fraction of absorbed dose recovered in urine as the studied metabolite was not considered for the purpose of comparing concentration-based dose estimates with doses reconstructed from cumulative amounts but this does not have any impact on the results of comparisons.

The validity of extrapolating nighttime excretions to 24-h excretions was verified by comparing the hourly excretion rate calculated on the basis of the nighttime collections to the hourly excretion rate of metabolites established on the basis of 24-h collections in one of the studied population samples (adults of study 1). The comparisons were performed using the non-parametric Wilcoxon test for related samples and the Spearman correlation test.

##### Estimation of daily absorbed dose from metabolite volume-weighted concentrations in nighttime collections

For dose reconstruction from volume-weighted concentrations (DDVWC_p_), the approach published by Fenske et al. [37] was followed (equation 3). Daily exposure to permethrin was determined from the volume-weighted concentrations (μg/L) by multiplying it by age- and gender-specific estimates of daily urinary volumes taken from the medical literature [44]. This daily exposure was calculated for the individuals of each studied population.

##### Estimation of daily absorbed dose from metabolite creatinine-adjusted concentrations in nighttime collections

For dose reconstruction from creatinine-adjusted concentrations (DDCAC_p_), the approaches published by Fenske et al. [37] and by Mage et al. [39] were followed (equation 4). Using the method of Fenske et al., daily exposure to permethrin was estimated by multiplying creatinine-adjusted concentrations (μg/g of creatinine) by daily creatinine excretions values taken from the medical literature [45]. Using the method of Mage et al., daily exposure to permethrin was estimated by multiplying creatinine-adjusted concentrations (μg/g of creatinine) by daily amounts of creatinine in urine calculated on the basis of formulas that account for gender, age, height and weight differences between participants.

(1) MHER = ([DCCA] × volume)/duration

(2) DDCA_p _= MHER × 24 h × MW_p_

(3) DDVWC_p _= [DCCA] × DUV × MW_p_

(4) DDCAC_p _= [DCCA]_cr _× DCE ÷ MW_DCCA _× MW_p_

Where: MHER = Metabolite excretion rate

DDCA_p _= Daily dose of permethrin calculated from cumulative amounts of metabolite in urine (μg/kg bw/day)

DDVWC_p _= Daily dose of permethrin calculated from volume-weighted concentration of metabolite in urine (μg/kg bw/day)

DDCAC_p _= Daily dose of permethrin calculated from creatinine-adjusted concentration of metabolite in urine (μg/kg bw/day)

[DCCA] = sum of molar concentrations (nmol/L) of the cDCCA and tDCCA (*cis/trans*) conformational isomers

[DCCA]_cr _= sum of creatinine-adjusted concentration (μg/g of creatinine) of cDCCA + tDCCA

volume = total urine volume over the collection period (L)

duration = time elapse between the last void prior to onset of collection and the last void collected in the bottle (h)

MW_p _= Molecular weight of permethrin. Permethrin was chosen since it appears as one of the main pyrethroids to which the general population is chronically exposed [4] (I. Gorse, personal communication].

MW_DCCA _= Molecular weight of DCCA

DUV = Daily urinary volume (L) taken from the medical literature [44]

DCE = Daily creatinine excretion (g) taken from the medical literature [45] or predicted from formulas [39]

#### Effect of variability in the urinary flow rate and creatinine excretion rate on the comparison in biomarker values between two populations

The effect of the inter-individual variability in the urinary flow rate or creatinine excretion rate on results of the comparison between two populations of biomarker data expressed in different units was also assessed. The pyrethroid metabolite levels between the urban and rural populations (studies 2 and 3) were compared on the basis of either volume-weighted concentrations, creatinine-adjusted concentration or, as a reference, excretion rates of pyrethroid metabolites adjusted for the body weight. A pre-analysis Kolmogorov-Smirnov normality test indicated that a non-parametric approach should be preferred because the volume-weighted concentrations, creatinine-adjusted concentrations and excretion rates of pyrethroid metabolites were not normally distributed. Comparison of the biomarker data between the urban and rural populations (adults and children separately) was thus performed using the non-parametric Mann-Whitney U test. Since the purpose of these comparisons was to assess the impact of the units of expression of biomarker data on results, samples below the detection limit were excluded. Differences in urinary flow rates and creatinine excretion rates between the urban and rural populations were also assessed using the Student t-test and Mann-Whitney U test, when the data was normally or not-normally distributed, respectively.

## Results

### Variability in the urinary flow rate and creatinine excretion rate

#### Intra-individual variability in urinary flow rate and creatinine excretion rate

Table 3 presents the intra-individual variability in the hourly urinary flow rate and creatinine excretion rate over a 24-h period, as assessed in the 15 subjects of study 1. Depending on the participant, the max/min ratio in the urinary flow rate (hence urine dilution) during a 24-h period varied between 2.5 and ≈ 18-fold. This corresponds to a coefficient of variation of 36.1 to 101%. Creatinine excretion rate was found to be less variable than the urinary flow rate. For a given individual, a 1.1 to 6.6-fold variation (max/min) in the creatinine excretion rate was observed over a 24-h period. This translates into a coefficient of variation of up to ≈ 48%.

**Table 3:** Intra-individual variability in hourly urinary flow rate and creatinine excretion rate over a 24-h period

	Urinary flow rate					Creatinine excretion rate				
		
	Mean (mL/h)	Min (mL/h)	Max (mL/h)	Max Min	CV^a^ %	Mean (g/h)	Min (g/h)	Max (g/h)	Max Min	CV^a^ %
Subject 1	66.9	38.4	96.5	2.5	36.1	0.074	0.043	0.10	2.4	32.9
Subject 2	67.7	38.4	107	2.8	48.2	0.061	0.055	0.069	1.3	9.66
Subject 3	120	24.9	437	18	101	0.044	0.031	0.051	1.7	13.7
Subject 4	119	57.6	176	3.1	42.3	0.038	0.030	0.047	1.6	14.5
Subject 5	68.8	23.2	123	5.3	57.0	0.027	0.012	0.038	3.2	32.0
Subject 6	46.2	24.7	62.4	2.5	42.0	0.035	0.028	0.044	1.6	24.0
Subject 7	91.0	22.9	185	8.1	77.7	0.034	0.026	0.040	1.5	14.3
Subject 8	53.9	26.9	107	4.0	68.5	0.069	0.065	0.074	1.1	6.06
Subject 9	38.0	20.3	57.3	2.8	40.0	0.052	0.049	0.055	1.1	6.00
Subject10	70.9	25.1	99.0	3.9	42.8	0.037	0.032	0.045	1.4	15.4
Subject 11	49.0	24.0	67.6	2.8	38.8	0.037	0.022	0.047	2.1	31.0
Subject 12	97.9	21.3	168	7.9	58.9	0.033	0.015	0.044	2.9	34.0
Subject 13	50.3	28.3	84.9	3.0	45.7	0.065	0.034	0.082	2.4	27.6
Subject 14	93.4	53.5	167	3.1	54.2	0.055	0.049	0.077	1.6	17.9
Subject 15	98.4	14.6	188	13	59.7	0.037	0.010	0.063	6.6	48.4

^a^CV = coefficient of variation (standard deviation/mean × 100%)

#### Inter-individual variability in urinary flow rate and creatinine excretion rate

Table 4 shows the inter-individual variability in the hourly urinary flow rate and creatinine excretion rate (calculated on the basis of an overnight collection from 6 pm until the next morning, including the first void on rising), as assessed for the infantile and adult urban population (study 2), rural population (study 3) and both populations pooled together (studies 2 and 3). In adults, there was up to an 18-fold inter-individual variation (max/min) in the hourly urinary flow rate, as compared to a 6.5-fold variation in children.

**Table 4:** Inter-individual variability in hourly urinary flow and creatinine excretion rates calculated from nocturnal urine collections

	Urinary flow rate					Creatinine excretion rate				
		
	Mean (mL/h)	Range (Min-Max) (mL/h)	Percentiles (5–95^th^) (mL/h)	Max Min	CV^a^ %	Mean (mL/h)	Range (Min-Max) (mL/h)	Percentiles (5–95^th^) (mL/h)	Max Min	CV^a^ %
**Adults**										
Urban (n = 74)	59.8	7.21–133	21.8–113	18	47.1	0.049	0.016–0.10	0.019–0.084	6.7	36.4
Rural (n = 109)	63.4	20.6–127	29.7–108	6.2	38.7	0.062	0.020–0.14	0.034–0.10	7.1	35.6
Both (n = 183)	62.0	7.21–133	26.4–112	18	42.1	0.057	0.016–0.14	0.029–0.095	9.2	37.4
**Children**										
Urban (n = 74)	32.0	10.7–69.5	13.4–61.9	6.5	48.0	0.027	0.011–0.082	0.014–0.045	7.7	42.1
Rural (n = 42)	26.6	10.8–64.6	14.3–48.7	6.0	45.9	0.025	0.0071–0.065	0.013–0.042	9.1	44.6
Both (n = 116)	30.1	10.7–69.5	13.6–61.4	6.5	48.2	0.026	0.0071–0.082	0.014–0.045	12	43.1

^a^CV = coefficient of variation (standard deviation/mean × 100%)

On the other hand, in adults, the highest creatinine excretion rate was 9.2 times higher than the smallest rate and in children there was a 12-fold inter-individual variation in the creatinine excretion rate, expressed in grams per hour.

**Figure 1:**
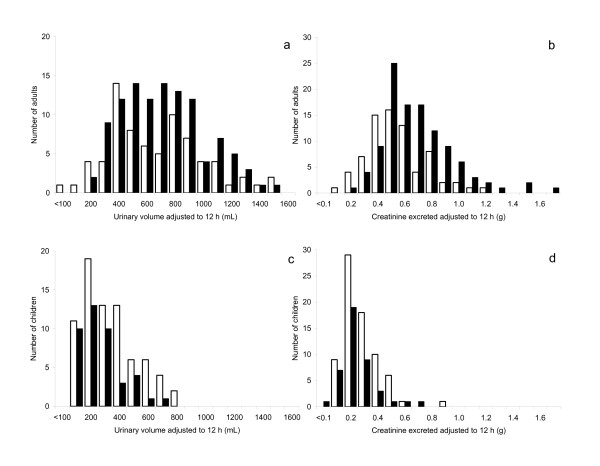
Urine volumes (mL/12 h) and creatinine amounts (g/12 h) in adults and children from urban (□) and rural (•) populations.

The corresponding variability in total volume and creatinine contents in adults and children over a normalized 12-h collection period is also depicted in Figure 1. The distributions observed in the urban and rural population were quite similar, except in the case of creatinine excretion in adults (Figure 1b) where the distribution in the rural population was displaced to the right compared to the urban population (Mann-Whitney p < 0.001).

### Effect of inter-individual variability in the urinary flow rate and creatinine excretion rate on individual exposure assessment

The impact of the units of expression of urinary biomarker measurements used to estimate individual absorbed daily dose of a pyrethroid was assessed in adults and children of urban and rural populations. It was first determined that daily urinary amounts of pyrethroid metabolites could be inferred from amounts in nighttime collections. The hourly excretion rate of DCCA and PBA in urines collected from 6 pm until the next morning including the first morning void was not significantly different from urinary metabolite hourly excretion rates calculated on the basis of 24-h urine collections (Wilcoxon p values = 0.177 for DCCA and 0.381 for PBA). A good correlation was also observed between the two variables (Spearman rho = 0.925 for DCCA and 0.934 for PBA (p values < 0.001)).

The estimated absorbed daily dose of permethrin reconstructed from volume-weighted concentrations (as proposed by Fenske et al. [37]) (Table 5) or creatinine-adjusted concentrations (as proposed by Fenske et al. [37] and Mage et al. [39]) (Tables 6 and 7) was then compared to the permethrin daily absorbed dose obtained directly from cumulative molar amounts of metabolites in overnight urine collections. In those tables, the data presented correspond to the estimates for the individuals with urinary flow rates and creatinine excretion rates equal or closest to the minimum, 5^th ^percentile, median, 95^th ^percentile and maximum of their respective population sample. The absorbed doses of permethrin estimated from volume-weighted concentrations of DCCA in the adults or children from the urban or rural area with a median urinary flow rate differed by 0 to 46% from the values calculated directly from cumulative amounts of biomarker in urine (Table 5). However, for the individuals with the lowest urinary output rate, doses reconstructed from volume-weighted concentrations of DCCA overestimated by 96 to 573%, hence by ≈ 2.0 to 6.7 times, the doses calculated from cumulative urinary amounts of biomarker. For the participants with the highest urinary flow rate, doses reconstructed from concentrations underestimated by 39 to 70%, hence by about 0.3 to 0.6 times, the values obtained from cumulative amounts. Correspondingly, when considering the 5 and 95^th ^percentile of urinary flow rates instead of the minimum and maximum values, an overestimation of 160% and an underestimation of 55% were calculated.

**Table 5:** Absorbed permethrin dose estimated from volume-weighted concentrations (V) and amounts (A) of DCCA

		Permethrin absorbed dose		
		
	Relative urinary flow rate	(A) Estimated from cumulative amounts (μg/kg bw/day)	(V) Estimated from volume-weighted concentrations^a ^(μg/kg bw/day)	V/A
**Adults**				
Urban				
	Lowest^b^	0.00354	0.0238	6.7
	5th percentile^c^	0.0564	0.134	2.4
	Median^d^	0.00941	0.0137	1.5
	95th percentile^e^	0.00571	0.00390	0.68
	Highest^f^	0.0304	0.0111	0.37
				
Rural				
	Lowest^b^	0.00143	0.00337	2.4
	5th percentile^c^	0.0113	0.0295	2.6
	Median^d^	0.00573	0.00725	1.3
	95th percentile^e^	0.00241	0.00108	0.45
	Highest^f^	0.0203	0.0123	0.61
				
**Children**				
Urban				
	Lowest^b^	0.0185	0.0365	2.0
	5th percentile^c^	0.00551	0.00833	1.5
	Median^d^	0.00537	0.00621	1.2
	95th percentile^e^	0.0310	0.0170	0.55
	Highest^f^	0.0258	0.00780	0.30
				
Rural				
	Lowest^b^	0.00481	0.00941	2.0
	5th percentile^c^	0.104	0.222	2.1
	Median^d^	0.00966	0.00970	1.0
	95th percentile^e^	0.0183	0.0115	0.63
	Highest^f^	0.0188	0.00980	0.52

^a^Calculated according to the method of Fenske et al. [37]

^b^Absorbed dose estimates for the participant with the lowest urinary flow rate

^c^Absorbed dose estimates for the participant with the urinary flow rate closest to the 5th percentile value

^d^Absorbed dose estimates for the participants with the median urinary flow rate

^e^Absorbed dose estimates for the participant with the urinary flow rate closest to the 95th percentile value

^f^Absorbed dose estimates for the participant with the highest urinary flow rate

**Table 6:** Absorbed permethrin dose estimated from creatinine-adjusted concentrations (C1) and amounts (A) of DCCA

		Permethrin absorbed dose		
		
	Relative creatinine excretion rate	(A) Estimated from cumulative amounts (μg/kg bw/day)	(C1) Estimated from creatinine-adjusted concentrations^a ^(μg/kg bw/day)	C1/A
**Adults**				
Urban				
	Lowest^b^	0.00625	0.0167	2.7
	5th percentile^c^	0.00749	0.0159	2.1
	Median^d^	0.835	0.739	0.89
	95th percentile^e^	0.0153	0.0131	0.86
	Highest^f^	0.00912	0.00622	0.68
				
Rural				
	Lowest^b^	0.0164	0.0340	2.1
	5th percentile^c^	0.0136	0.0167	1.2
	Median^d^	0.0155	0.0191	1.2
	95th percentile^e^	0.0104	0.00722	0.69
	Highest^f^	0.0286	0.0142	0.50
				
**Children**				
Urban				
	Lowest^b^	0.0185	0.0469	2.5
	5th percentile^c^	0.177	0.342	1.9
	Median^d^	0.0120	0.00667	0.56
	95th percentile^e^	0.00754	0.00461	0.61
	Highest^f^	0.0818	0.0137	0.17
				
Rural				
	Lowest^b^	0.00481	0.00932	1.9
	5th percentile^c^	0.00631	0.00670	1.1
	Median^d^	0.0155	0.0189	1.2
	95th percentile^e^	0.0237	0.0153	0.65
	Highest^f^	0.0188	0.00786	0.42

^a^Calculated according to the method of Fenske et al. [37]

^b^Absorbed dose estimates for the participant with the lowest creatinine excretion rate

^c^Absorbed dose estimates for the participant with the creatinine excretion rate closest to the 5th percentile value

^d^Absorbed dose estimates for the participants with the median creatinine excretion rate

^e^Absorbed dose estimates for the participant with the creatinine excretion rate closest to the 95th percentile value

^f^Absorbed dose estimates for the participant with the highest creatinine excretion rate

**Table 7:** Absorbed permethrin dose estimated from creatinine-adjusted concentrations (C2) and amounts (A) of DCCA

		Permethrin absorbed dose		
		
	Relative creatinine excretion rate	(A) Estimated from cumulative amounts (μg/kg bw/day)	(C2) Estimated from creatinine-adjusted concentrations^a ^(μg/kg bw/day)	C2/A
**Adults**				
Urban				
	Lowest^b^	0.00625	0.0120	1.9
	5th percentile^c^	0.00749	0.0145	1.9
	Median^d^	0.835	0.807	0.97
	95th percentile^e^	0.0153	0.0136	0.89
	Highest^f^	0.00912	0.00716	0.79
				
Rural				
	Lowest^b^	0.0164	0.0313	1.9
	5th percentile^c^	0.0136	0.0155	1.1
	Median^d^	0.0155	0.0170	1.1
	95th percentile^e^	0.0104	0.00730^g^	0.73
	Highest^f^	0.0286	0.0103^g^	0.36
				
**Children**				
Urban				
	Lowest^b^	0.0185	0.0256	1.4
	5th percentile^c^	0.177	0.5103	2.9
	Median^d^	0.0120	0.0113	0.94
	95th percentile^e^	0.00754	0.00465	0.62
	Highest^f^	0.0818	0.0315	0.39
				
Rural				
	Lowest^b^	0.00481	0.00674	1.4
	5th percentile^c^	0.00631	0.00650	1.0
	Median^d^	0.0155	0.0144	0.93
	95th percentile^e^	0.0237	0.0176	0.74
	Highest^f^	0.0188	0.0158	0.84

^a^Calculated according to the method of Mage et al. [42]

^b^Absorbed dose estimates for the participant with the lowest creatinine excretion rate

^c^Absorbed dose estimates for the participant with the creatinine excretion rate closest to the 5th percentile value

^d^Absorbed dose estimates for the participants with the median creatinine excretion rate

^e^Absorbed dose estimates for the participant with the creatinine excretion rate closest to the 95th percentile value

^f^Absorbed dose estimates for the participant with the highest creatinine excretion rate

^g^This individual was obese (BMI > 30.0) and, in this specific case, Mage et al. [42] suggest to use lean body mass for the prediction of creatinine excretion

The absorbed doses of permethrin estimated from creatinine-adjusted concentrations (method of Fenske et al. [37]) of DCCA in adults and children of the urban or rural population with median creatinine excretion rates differed by -44% to +23% from the doses calculated directly from cumulative urinary amounts (Table 6). For the participants with creatinine excretion rates closest to the 5^th ^and 95^th ^percentiles of their population sample, the error ranged from -39% to +112%. For the participants with the lowest creatinine excretion rate, doses reconstructed from creatinine-adjusted concentrations overestimated by 94 to 167%, hence by 2 to 3 times, the doses calculated directly from cumulative urinary amounts of biomarker. For the individuals with the highest creatinine excretion rate, doses reconstructed from creatinine-adjusted concentrations underestimated by 32% to more than 83%, hence by 0.2 to 0.7 times, the values obtained from cumulative amounts. With a more refined approach published by Mage et al. [39] for dose reconstruction based on creatinine-adjusted concentrations that takes into account the height of the subjects in addition to their weight and age, the estimates of absorbed dose were slightly closer to the values calculated from cumulative amounts of biomarker (Table 7), ranging from -63% to +93% of the actual amounts in the case of adults and from -61% to +187% in the case of children.

### Effect of inter-individual variability in the urinary flow rate and creatinine excretion rate on the comparison of biomarker values between two populations

Table 8 shows the effect of the units of measurements (volume-weighted or creatinine adjusted concentrations) on results of the comparison of pyrethroid biomarker levels between two populations (urban versus rural). These results were compared to the reference ones obtained when assessing the difference between the two populations in hourly excretion rate values of pyrethroid metabolites normalized per unit of body weight (μg/h/kg bw). Comparison conducted on the basis of volume-weighted concentrations of DCCA or PBA lead to results similar to those obtained on the basis of excretion rates of these metabolites.

**Table 8:** Mann-Whitney comparisons of metabolite levels, expressed in different units, between urban and rural populations

Population and metabolite assessed	Statistical significance (p value) of comparisons between the urban and rural population		
	
	Comparison of weight-adjusted excretion rates (nmol/kg bw/h)	Comparison of volume-weighted concentrations (nmol/L)	Comparison of creatinine-adjusted concentrations (μmol/mol of creatinine)
**Adults**			
DCCA	0.128	0.139	0.465
PBA	0.016*	0.010*	0.093
**Children**			
DCCA	0.032*	0.042*	0.029*
PBA	0.027*	0.023*	0.023*

*Indicates a significant difference

Comparison of pyrethroid levels between the urban and rural adult population on the basis of creatinine-adjusted DCCA and PBA concentrations lead to more divergent results. In particular, a non significant difference in PBA creatinine-adjusted concentrations was observed between the two groups contrary to what was observed on the basis of PBA excretion rates or volume-weighted concentrations. It was observed that the rural adult population had a significantly higher creatinine excretion rate than the urban population (p < 0.001), which may have biased the test, whereas the distribution of the urinary flow rate was similar between the two groups (p = 0.357). In children, no significant difference in either the urinary flow rate or creatinine excretion rate was observed between the urban and rural population (p = 0.081 and 0.160, respectively).

## Discussion

The purpose of this research was to document the intra- and inter-individual variability in creatinine excretion rate and urinary flow rate in order to determine the relative impact of the different units of expression of pyrethroid biomarker data i) on reconstructed absorbed doses in individuals and ii) on results of comparisons of population biomonitoring data. This led to identify the most suitable units of expression of biomonitoring data and straightforward strategies for data collection in the case of non-persistent pesticides such as pyrethroids.

Intra-individual day-to-day variations in daily creatinine excretion (g/day) have been well documented [46,47] but, to our knowledge, this is the first report of between-void variability in creatinine excretion rate and urinary flow rate during the course of a day. In an assessment of intra-individual day-to-day variations in creatinine excretion rate in 24-h urine collections based on several studies, Garde et al. [48] reported a coefficient of variation of 9–24%. However, void-to-void variations in creatinine excretion rate were not studied. Our findings demonstrate an important intra-individual variability in both the urinary flow rate (CV of 36.1 to 101%; max/min ratio of up to 18 fold) and creatinine excretion rate (CV of 6.0 to 48.4%; max/min ratio of up to 6.6. fold) during the course of a day in adults, the latter being larger than the day-to-day variations reported by Garde et al. [48]. Those variations are likely the consequence of fluid intake for the former and dietary protein intake [36] and physical activity [49] for the latter. It has also been reported that variations in the urinary flow rate may explain as much as 21% of the creatinine variability [50], although this finding has been disputed. Our results suggest that there could be a difference of nearly a factor seven in creatinine-adjusted concentrations of a metabolite measured in two different urine samples taken the same day from a same individual solely due to the intra-individual intra-day variation in creatinine excretion rate.

Inter-individual variability in the urinary output and creatinine excretion rate has also been extensively studied; factors influencing these variations include age, gender, ethnicity, fat-free mass and height, ambient temperature, physical activity [31,51,52]. Garde et al. [48] reported coefficient of variations for inter-individual variations in creatinine excretion rate in 24-h urine collections ranging from 17–25%. In our study, using a shorter urine collection period (over a ± 12-h nighttime collection), inter-individual variability in the urinary flow rate and creatinine excretion rate was further evaluated in 183 adults and 116 children from urban and rural areas. Creatinine excretion rate was found to be almost as variable (CV of 35.6 to 44.6%) as the urine volume (CV of 38.7–48.2%). This corresponds to a possible 9-fold (in adults) or 12-fold (in children) variation in the creatinine-adjusted concentrations of a metabolite solely due to the inter-individual variability in the creatinine-excretion rate. For urinary output, it implies that, if all adults absorbed the same dose of parent-compound, one could observe an 18-fold variation in metabolite concentrations solely due to inter-individual variations in the urine dilution. In children, the ratio between the more diluted and the more concentrated urines was about 6.

Results of our study show that variability in creatinine excretion and urinary output will directly influence the estimates of reconstructed absorbed dose of pyrethroids in an individual conducted on the basis of the volume-weighted or creatinine-adjusted concentrations of biomarkers. In 2007, Mage et al. [39] proposed a formula to predict daily creatinine excretion to estimate pesticide daily absorbed doses from creatinine-adjusted concentrations of metabolites in spot urine samples while accounting for inter-individual differences in age, gender, height and weight. Although this latter approach yields results closer to the cumulative amounts directly measured in urine than the approach used by Fenske et al. [37], it is nonetheless not very precise for individuals with marginal creatinine excretions (high or low), or atypical creatinine excretion (*i.e. *urinary creatinine excretion not well predicted by the formula), as observed in our study. In fact, errors in dose estimates based on creatinine-adjusted or volume-weighted concentrations can reach 500% or more in some cases.

It is to be noted that, in our estimation of the absorbed dose, the fraction of the dose recovered in urine as metabolites was not considered in dose reconstruction. This had no effect on our comparison of the different approaches for dose reconstruction but absolutely needs to be considered for accurate dose reconstruction and comparison with acceptable daily intakes.

With regard to the comparison of pyrethroid metabolite levels between two populations however, volume-weighted concentrations gave similar results to those obtained on the basis of cumulative amounts while discrepancies were observed between creatinine-adjusted concentrations and hourly excretion rates of some pyrethroid metabolites. When the urinary flow rates or creatinine excretion rates are normally distributed and not significantly different from one population to the other, comparison of metabolite levels between two populations may give similar results whatever the unit of measurement (volume-weighted concentration, creatinine-adjusted concentration or cumulative amounts). Conversely, if a significant difference in the urinary output or in the creatinine excretion rate is observed, then discrepancies in the comparison results may be observed depending on the units of measurements.

For instance, adults from the rural population were heavier than the urban adults, and possibly more physically active, which may have affected their creatinine excretion rate, hence results of comparisons of creatinine-adjusted concentrations of metabolites between the two population samples. In another context, if one would compare concentrations of a pyrethroid metabolite in an Asian population to those of North-Americans on the basis of creatinine-adjusted values, significant differences could emerge due to variations in creatinine excretion rate as a result of differences in muscular mass between the two populations (higher stature of North-Americans compared to Asians). Barr et al. [31] described in details the factors influencing inter-individual creatinine variability in the US population and analyzed the influence of different factors, including ethnicity, gender and age, with a multiple linear regression model. They then stratified creatinine concentrations in their population (n = 22 245, NHANES) according to these factors, thus providing reference values for different subgroups of the US population. The application of the regression model elaborated by Barr et al. [31] in such a context might not be adequate since the coefficients were derived for people of different races, but living more or less in the same environment. Therefore, to verify adequately that there is no bias in the results of comparisons of exposure levels assessed on the basis of volume-weighted or creatinine-adjusted concentrations between populations from different countries, one also needs to verify if there is a significant difference in the urinary flow rate or in the creatinine excretion rate, which requires timed collections. With timed-collections, metabolite excretion rate adjusted for the body weight can be directly determined and used for comparison of exposure between two populations instead of volume-weighted or creatinine-adjusted concentrations.

## Conclusion

Overall, although it is more practical in field studies to determine concentrations of non-persistent pesticide metabolites in spot urine samples and adjust for creatinine contents, our results show that it may lead to serious errors in the estimation of the actual daily absorbed doses, particularly at the individual level. For a more accurate assessment of individual absorbed doses, we conclude that it is better to determine total amounts of metabolites in urine collected over the longest feasible and practical period of time. Collection of overnight urine voids (6 pm to first morning void) limits the risk of urine loss and participant attrition that increases with longer urine collection periods and allows a valid assessment of the 24-h excretion rate of pyrethroid metabolites.

## Abbreviations

cDCCA: cis-3-(2,2-dichlorovinyl)-2,2-dimethylcyclopropane carboxylic acid; tDCCA: trans-3-(2,2-dichlorovinyl)-2,2-dimethylcyclopropane carboxylic acid; [DCCA]: sum of molar concentrations (nmol/L) of the cDCCA and tDCCA (*cis/trans*) conformational isomers; CV: coefficient of variation; %CV: coefficient of variation expressed as a percentage; [DCCA]_cr_: sum of creatinine-adjusted concentration (μg/g of creatinine) of cDCCA + tDCCA; DCE: daily creatinine excretion (g); DDCA_p_: daily dose of permethrin calculated from cumulative amounts of metabolite in urine (μg/kg bw/day); DDCAC_p_: daily dose of permethrin calculated from creatinine-adjusted concentration of metabolite in urine (μg/kg bw/day); DDVWC_p_: daily dose of permethrin calculated from volume weighted concentration of metabolite in urine (μg/kg bw/day); DUV: daily urinary volume (L); MAX: maximum; MAX/MIN: maximum to minimum ratio; MIN: minimum; MHER: metabolite excretion rate; MW_DCCA_: molecular weight of DCCA; MW_p_: molecular weight of permethrin; OP: organophosphate; PBA: 3-phenoxybenzoic acid; $\bar{\text{x}}$: mean.

## Competing interests

The authors declare that they have no competing interests.

## Authors' contributions

MCF conceived the study and its design, carried out the analysis, interpreted the results and drafted the manuscript. MB conceived the study and its design, interpreted the results and contributed to draft the manuscript. GC participated in the interpretation of the results. All authors read and approved the final manuscript.
